# Use of prehospital qSOFA in predicting in-hospital mortality in patients with suspected infection: A retrospective cohort study

**DOI:** 10.1371/journal.pone.0216560

**Published:** 2019-05-07

**Authors:** Satoshi Koyama, Yutaka Yamaguchi, Koichiro Gibo, Izumi Nakayama, Shinichiro Ueda

**Affiliations:** 1 Department of Emergency Medicine, Okinawa Chubu Hospital, Uruma, Japan; 2 Department of Clinical research and Education, University of the Ryukyus, Nishihara, Nakagami, Japan; 3 Intensive Care Unit, Department of Internal Medicine, Okinawa Chubu Hospital, Uruma, Japan; University of Colorado Denver, UNITED STATES

## Abstract

**Background:**

The quick sequential organ failure assessment (qSOFA) score has recently been introduced to the emergency department (ED) and wards, and it predicted a higher number of deaths among patients with sepsis compared with baseline risk. However, studies about the application of the qSOFA score are limited in prehospital settings. Thus, this study aimed to assess the performance of prehospital qSOFA score in predicting the risk of mortality among patients with infection.

**Methods:**

This single center, retrospective cohort study was conducted in a Japanese tertiary care teaching hospital between April 2016 and March 2017. We enrolled all consecutive adult patients transported to the hospital by ambulance and admitted to the ED due to a suspected infection. We calculated the prehospital qSOFA score using the first vital sign obtained at the scene by emergency medical service (EMS) providers. The primary outcome was in-hospital mortality. The Cox proportional hazards model was used to assess the association between prehospital qSOFA positivity and in-hospital mortality.

**Results:**

Among the 925 patients admitted to the ED due to a suspected infection, 51.1% (473/925) were prehospital qSOFA-positive and 48.9% (452/925) were prehospital qSOFA-negative. The in-hospital mortality rates were 14.0% (66/473) in prehospital qSOFA-positive patients and 6.0% (27/452) in prehospital qSOFA-negative patients. The Cox proportional hazard regression model revealed a strong association between prehospital qSOFA score and in-hospital mortality (adjusted hazard ratio: 2.41, 95% confidence interval: 1.51–3.98; p <0.01).

**Conclusions:**

Among the patients with suspected infection who were admitted at the ED, a strong association was observed between the prehospital qSOFA score and in-hospital mortality. In order to use this score in clinical practice, future study is necessary to evaluate how infection is suspected in the prehospital arena.

## Introduction

Sepsis is a global health burden with high prevalence and mortality rates. The time intervals from the first medical contact to the diagnosis of sepsis and the initiation of treatments, particularly the rapid administration of antibiotic, were associated with lower mortality rates [1–4]. For an accurate and early identification of patients suspected with sepsis and for the improvement of patient outcomes, an international task force of experts redefined sepsis syndrome and introduced the quick sequential (sepsis-related) organ failure assessment (qSOFA) score for non-intensive care unit (ICU) setting in 2016 [5]. Seymour et al. and Sepsis-3 Task Force have reported that qSOFA in the emergency department (ED) and wards predicted a higher number of deaths compared with baseline risk [2], and they recommended that the “qSOFA criteria should be used outside of the ICU to prompt clinicians to further investigate for organ dysfunction and to initiate or escalate therapy as appropriate.”

Prehospital care of sepsis has attracted broad attention. Like prehospital interventions for other time-sensitive conditions, including cardiac arrest, acute myocardial infarction, and stroke, prehospital care of sepsis is a promising intervention that improves outcomes [6–9]. Several prehospital screening tools used for the identification of sepsis have been developed before the introduction of the qSOFA score. However, they lack accuracy [10–14]. As in the EDs and wards, the qSOFA score may be used in prehospital setting because of its simple scoring system that uses only vital signs. However, studies that have investigated the relationship between qSOFA in the prehospital setting and patient outcomes are limited [15–17].

To address the knowledge gap in the literature, we calculated the prehospital qSOFA score using the first vital sign obtained at the scene by emergency medical service (EMS) providers and investigated the association between prehospital qSOFA score and in-hospital mortality in our community. This study aimed to assess the performance of prehospital qSOFA score in predicting the risk of mortality among patients with infection.

## Materials and methods

### Ethics approval and consent to participate

The institutional review board of Okinawa Chubu Hospital approved the study protocol (H30-90). Because of the retrospective nature of this study and the de-identification of personal data, the board waived the need for informed consent.

### Study design, setting, and patients

This was an observational study conducted at Okinawa Chubu Hospital, a tertiary care teaching hospital with 550 hospital beds and 14 ICU beds in Japan, between April 2016 and March 2017. We accepted patients who were transported from six EMS agencies in our district, with a population of approximately 460,000.

We examined the data of all adult (≥ 18 years) patients who were admitted to the ED by EMS agencies and were registered with the diagnosis name of an *International Classification of Diseases*, *Tenth Revision* (ICD-10) code indicative of infection (A00-B99, certain infectious and parasitic diseases; G00-05, neurologic infection; I30-32 and J38-40, endo/myocarditis; J00-06, J09-18, J20-22, J36, J40, and J85-86, respiratory infection; K35-37, appendicitis; K57, diverticulitis; K61, K63, K65, and K67, peritonitis and intestinal abscess; K75.0, liver abscess; K81 and K83, cholecystitis and cholangitis; L00-08, skin and soft tissue infections; M00-03 and M86, infective arthritis and osteomyelitis; N10 and N30, urinary tract infection; and N70-76, inflammatory disease of the female pelvic organ) on the electronic medical record between April 2016 and March 2017. In addition, because infection and sepsis are often undercoded, we also assessed for patients who received antibiotic treatments during their hospital stay.

We excluded patients whose EMS records were missing. Patients who did not receive antibiotics within 48 hours after ED arrival were also excluded because they were less likely to have severe bacterial infection. The other exclusion criterion was patients who had do-not-resuscitate (DNR) code prior to ED admission.

### Data collection

The demographic information of the patients and related characteristics were obtained from in-hospital electronic medical records and paper-based EMS records. We collected data for analyses, which included age, sex, comorbidities, use of immunosuppressants, location prior to ED admission, prehospital and ED triage vital signs, laboratory data, primary site of infection, type of organisms, ICU admission, length of ICU stay, length of hospital stay, and prevalence of bacteremia and in-hospital mortality. We checked the presence of comorbidities that were categorized in the Charlson comorbidity index (CCI) [18]. Laboratory data included leucocyte count, hematocrit levels, platelet count, PT-INR, and serum sodium, potassium, CRP, glucose, and lactate levels. The primary site of infection was diagnosed by means of confirmation via clinical, radiological, and microbiological examinations. The type of organisms was determined based on various culture results. Bacteremia was diagnosed if we detected the same microorganisms from two sets of blood culture bottles.

### Measurement of the primary exposure factors

The qSOFA score had three criteria: assigning one point for alteration in mental status (Glasgow coma scale [GCS] score <15), systolic blood pressure ≤ 100 mmHg, and respiratory rate ≥ 22/min, respectively. We calculated the prehospital qSOFA score using the first vital sign obtained at the scene by EMS providers. If the vital sign was not recorded at the scene, we adopted the first vital sign en route instead. For the prehospital evaluation of mental status, the Japanese EMS providers have adopted the Japan coma scale (JCS) instead of the GCS since its introduction in 1974 [19]. JCS has four main grades (grade 0: alert; grade 1: possible verbal response without any stimulation, not lucid; grade 2: possible eye-opening, verbal and motor response upon stimulation; and grade 3: no eye-opening and coma upon stimulation). Therefore, we count JCS grades 1, 2, and 3 as one point of the qSOFA for alteration in mental status. According to previous studies, we defined prehospital qSOFA positivity or negativity as the prehospital qSOFA score ≥ 2 or < 2, respectively [2, 5].

### Outcome measures

The primary outcome measure was in-hospital all-cause mortality. The secondary outcomes were 28- and 90-day mortality as confirmed by follow-up visits after discharge.

### Statistical analysis

Continuous data were presented as medians with interquartile range (IQR) and were compared using the Mann-Whitney U test. Categorical data were presented as proportions and were compared using Fisher’s exact test when appropriate. We used the Kaplan-Meier plots to describe the survival of prehospital qSOFA-positive and qSOFA-negative patients and to compare the survival curves with the log-rank test. Moreover, the Cox proportional hazards model was used in assessing the association between the prehospital qSOFA positivity and in-hospital mortality censored during the discharge day and 28 and 90 days after ED admission after adjusting for other risk factors of mortality. Based on a priori knowledge, the following variables were incorporated into the primary multivariable models: age, sex, presence of chronic health condition, and location prior to ED admission. We defined chronic health condition as congestive heart failure, dementia, chronic pulmonary disease, rheumatologic disease, mild liver disease, diabetes with complications, hemiplegia, renal disease, hematologic malignancy, moderate or severe liver disease, metastatic solid tumor, and AIDS/HIV, which were comorbidities of the CCI associated with prognosis [20, 21]. We considered the use of immunosuppressants as chronic health condition because it increases the risk of infection.

A subgroup analysis of in-hospital mortality was conducted to validate the interaction between subgroup factors and the prehospital qSOFA score. Subgroups were defined by age, sex, presence of chronic health condition, location prior to ED admission, and site of infection. We set the age threshold to 75 years or over according to the definition of elderly individuals in Japan. The site of infection was categorized as respiratory or other sites in accordance to prior study [22]. We used the Cox proportional hazard model for analyses after adjusting the same covariates used in the main group analysis except for the variable for stratification. All statistical analyses were performed using R (The R Foundation for Statistical Computing, ver. 3.2.4) and JMP Pro software (ver. 1.31, SAS Institute Inc., Cary, NC, the USA). All tests were two-tailed; p values < 0.05 were considered statistically significant.

## Results

**Fig 1:**
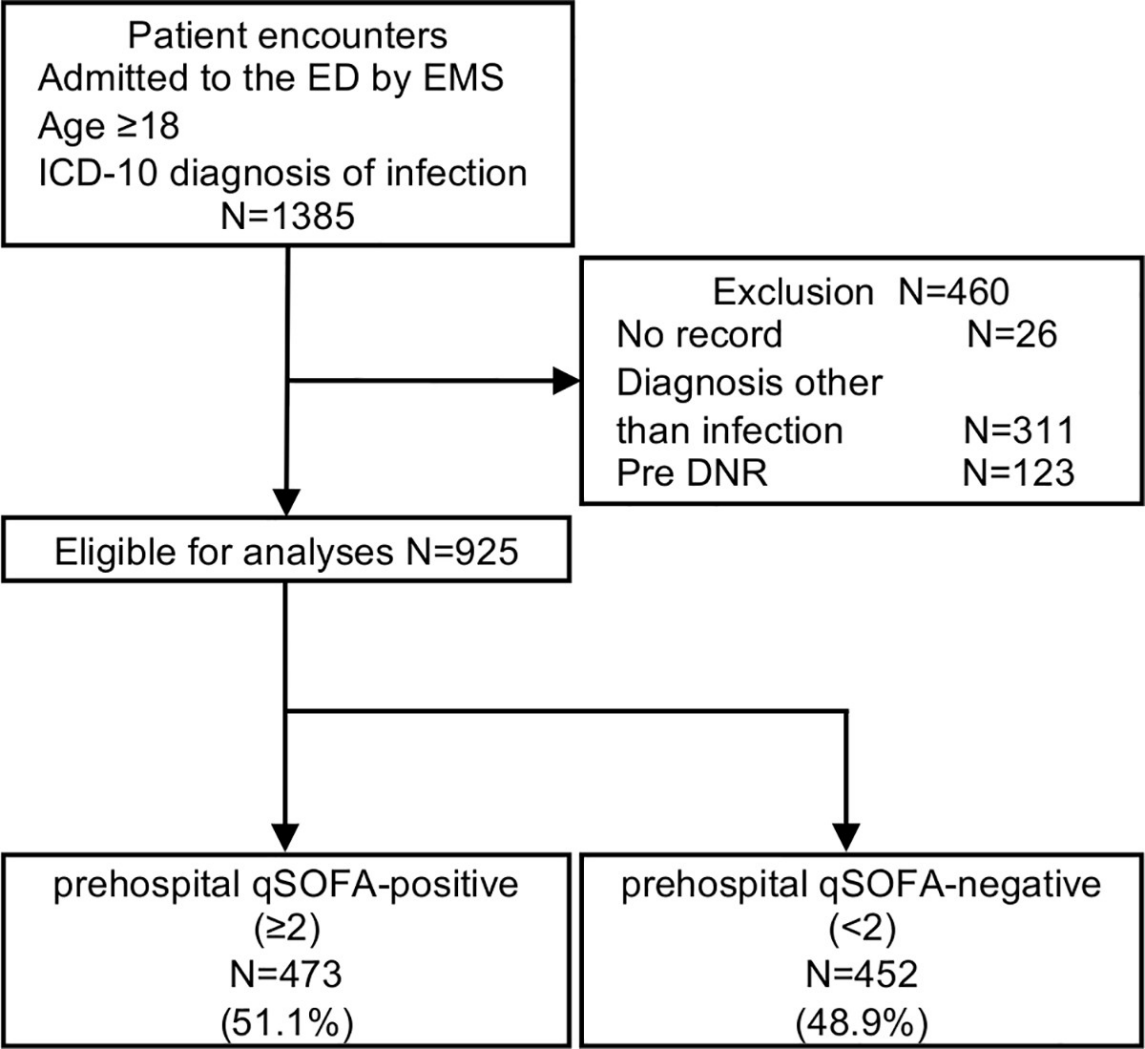
Flow diagram of patient recruitment. Pre DNR suggests patients who had do-not-resuscitate (DNR) code prior to ED admission. Abbreviations: ED, emergency department; EMS, emergency medical service; ICD-10, International Classification of Diseases, Tenth Revision; DNR, do-not-resuscitate; qSOFA, quick sequential organ failure assessment.

The flow diagram of patient recruitment is shown in Fig 1. From April 2016 to March 2017, a total of 1385 patients were admitted to the ED due to a suspected infection. Of these patients, 460 were excluded due to the following reasons: missing EMS record, lack of antibiotic treatment within 48 hours of ED arrival, and assignment of DNR code prior to ED admission. Finally, 925 patients were enrolled for our analyses. Among the 925 patients, 51.1% (473/925) and 48.9% (452/925) were positive and negative for prehospital qSOFA, respectively.

The demographic information of patients, characteristics at presentation, and hospital course after ED admission are summarized in Table 1 and S1 Table. Prehospital qSOFA-positive patients were slightly older than prehospital qSOFA-negative patients (prehospital qSOFA-positive: 82 [IQR 71–88] vs prehospital qSOFA-negative: 79 [IQR 67–86], p<0.01) and a higher number of patients were from a nursing home (prehospital qSOFA-positive: 40.6% [192/473] vs prehospital qSOFA-negative: 18.6% [84/452], p<0.01). No difference was observed between the two groups in terms of sex. Hemiplegia (prehospital qSOFA-positive: 27.9% [132/473] vs prehospital qSOFA-negative: 13.3% [60/452], p<0.01) and dementia (prehospital qSOFA-positive: 21.4% [101/473] vs prehospital qSOFA-negative: 10.8% [49/452], p<0.01) were observed more frequently in prehospital qSOFA-positive patients than in prehospital qSOFA-negative patients. Body temperature was similar between the two groups. Laboratory results were similar except for PT-INR (prehospital qSOFA-positive: 1.13 [IQR: 1.04–1.25] vs prehospital qSOFA-negative: 1.08 [IQR: 1.02–1.18], p<0.01), serum sodium levels (prehospital qSOFA-positive: 135 [IQR: 131–139] vs prehospital qSOFA-negative: 136 [IQR: 132–138], p<0.05), serum potassium levels (prehospital qSOFA-positive: 4.1 [IQR: 3.7–4.5] vs prehospital qSOFA-negative: 3.9 [IQR: 3.5–4.3], p<0.01), and lactic acid levels (prehospital qSOFA-positive: 1.8 [IQR: 1.1–3.3] vs prehospital qSOFA-negative: 1.5 [IQR: 1.1–2.2], p<0.01) between the two groups. Site of infection, type of organisms, and bacteremia did not significantly differ between the two groups. The number of prehospital qSOFA-positive patients who were admitted in the ICU was slightly higher than that of prehospital qSOFA-negative patients. However, no significant difference was observed between the two groups (prehospital qSOFA-positive: 11.0% [52/473] vs prehospital qSOFA-negative: 8.6% [39/452], p = 0.269).

**Table 1:** Demographic data and characteristics of patients.

	prehospital qSOFA	prehospital qSOFA	
	negative (<2)	positive (≥2)	
	(N = 452)	(N = 473)	p value
Age (median [IQR])	79 [67, 86]	82 [71, 88]	<0.01
Male (%)	227 (50.2)	242 (51.2)	0.79
**Comorbidities (%)**			
Diabetes with complication	35 (7.7)	24 (5.1)	0.11
Congestive heart failure	14 (3.1)	17 (3.6)	0.72
Chronic pulmonary disease	46 (10.2)	46 (9.7)	0.83
Mild liver disease	11 (2.4)	9 (1.9)	0.65
Moderate-severe liver disease	13 (2.9)	12 (2.5)	0.84
Renal disease	20 (4.4)	20 (4.2)	1.00
Rheumatologic disease	20 (4.4)	24 (5.1)	0.76
Hemiplegia or paraplegia	60 (13.3)	132 (27.9)	<0.01
Dementia	49 (10.8)	101 (21.4)	<0.01
Hematologic malignancy	8 (1.8)	14 (3.0)	0.28
Metastatic solid tumor	17 (3.8)	18 (3.8)	1.00
AIDS/HIV	1 (0.22)	0 (0)	0.49
Immunosuppressant	39 (8.6)	27 (5.7)	0.10
**Location (%)**			<0.01
Home	314 (69.5)	232 (49.1)	
Nursing home	84 (18.6)	192 (40.6)	
Medical faculty	54 (12.0)	49 (10.4)	
**Prehospital vital (median [IQR])**			
Systolic blood pressure, mmHg	130 [120, 150]	120 [100, 140]	<0.01
Heart rate, /min	98 [84, 112]	102 [88, 120]	<0.01
Respiratory rate, /min	24 [20, 30]	30 [24, 32]	<0.01
Japan Coma Scale	0 [0, 0]	1 [1, 2]	<0.01
Glasgow Coma Scale	15 [15, 15]	10 [8, 13]	<0.01
Body temperature, Celsius	37.7 [36.9, 38.6]	37.9 [37.0, 38.9]	<0.01
**ED triage vital (median [IQR])**			
Systolic blood pressure, mmHg	134 [120, 150]	120 [100, 140]	<0.01
Heart rate, /min	98 [85, 110]	103 [88, 120]	<0.01
Respiratory rate, /min	22 [20, 27.75]	24 [20, 30]	<0.01
Glasgow Coma Scale	15 [13, 15]	11 [9, 14]	<0.01
Body temperature, Celsius	37.7 [37.0, 38.6]	37.9 [37.0, 38.9]	0.29
Bacteremia (%)	75 (16.6)	81 (17.1)	0.86
ICU Admission (%)	39 (8.6)	52 (11.0)	0.27
ICU LOS (median [IQR])	0 [0, 0]	0 [0, 0]	0.24
Hospital LOS (median [IQR])	11 [7, 19]	12 [8, 18.5]	0.56

The prehospital qSOFA score was assessed using the first vital sign obtained at the scene and was taken by EMS providers.

Abbreviation: qSOFA, quick sequential organ failure assessment; IQR, interquartile range; ED, emergency department; ICU, intensive care unit; LOS, length of stay; EMS, emergency medical service.

**Fig 2:**
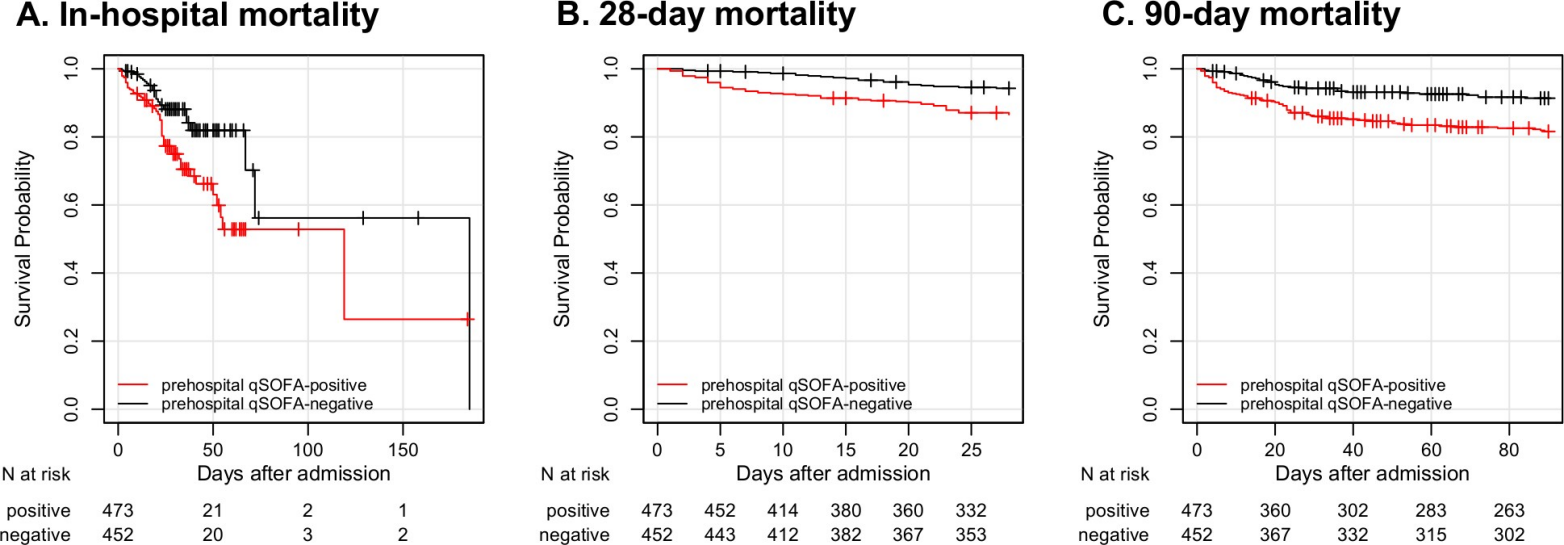
Kaplan-Meier curves stratified as prehospital qSOFA-positive or prehospital qSOFA-negative. A, In-hospital mortality censored at the discharge day. B, 28-day mortality censored 28 days after ED admission. C, 90-day mortality censored 90 days after ED admission. The vertical tick marks on the curves represent censoring due to survival discharge. p value < 0.01 (log-rank test). Abbreviation: qSOFA, quick sequential organ failure assessment; ED, emergency department.

The primary and secondary outcomes are shown in Fig 2 and Table 2. Overall, the in-hospital mortality rate was 10.1% (93/925), and 14.0% (66/473) of prehospital qSOFA-positive patients died compared to 6.0% (27/452) of prehospital qSOFA-negative patients on discharge day. With regard to the secondary outcomes, 15.9% (61/384) of prehospital qSOFA-positive patients died compared to 6.3% (23/363) of prehospital qSOFA-negative patients 28 days after ED admission, and 24.4% (83/340) of prehospital qSOFA-positive patients died compared to 10.3% (34/329) of prehospital qSOFA-negative patients 90 days after ED admission. The Kaplan-Meier plots of survival showed a significant difference between the two groups (p <0.001). The Cox proportional hazard regression model revealed that prehospital qSOFA positivity has a strong association with in-hospital mortality (unadjusted hazards ratio [HR]: 2.45, 95% confidence interval [CI]: 1.58–3.92; p <0.01). After adjusting for confounders (age, gender, chronic health condition, and location prior to ED admission), prehospital qSOFA positivity still has a strong association with in-hospital mortality (adjusted HR: of 2.41, 95% CI: 1.51–3.98; p <0.01).

**Table 2:** Unadjusted & adjusted hazard ratios for mortality in patients who were positive for prehospital qSOFA.

	HR (95% CI)	p value
**In-hospital mortality**		
Unadjusted	2.45 (1.58–3.92)	<0.01
Adjustment 1	2.39 (1.53–3.84)	<0.01
Adjustment 2	2.51 (1.58–4.13)	<0.01
Adjustment 3	2.41 (1.51–3.98)	<0.01
**28-day mortality**		
Unadjusted	2.55 (1.60–4.22)	<0.01
Adjustment 1	2.48 (1.55–4.10)	<0.01
Adjustment 2	2.58 (1.59–4.32)	<0.01
Adjustment 3	2.39 (1.46–4.03)	<0.01
**90-day mortality**		
Unadjusted	2.36 (1.58–3.59)	<0.01
Adjustment 1	2.30 (1.54–3.51)	<0.01
Adjustment 2	2.50 (1.65–3.86)	<0.01
Adjustment 3	2.35 (1.54–3.65)	<0.01

The primary analysis was performed with the Cox proportional hazard regression model and in-hospital mortality censored at the discharge day and 28 and 90 days after ED admission.

Adjustment 1 was for the demographic characteristics of patients (age and sex).

Adjustment 2 was for the demographic characteristics of patients, as previously mentioned, and presence of any chronic health condition (congestive heart failure, dementia, chronic pulmonary disease, rheumatologic disease, mild liver disease, diabetes with complications, hemiplegia, renal disease, hematologic malignancy, moderate or severe liver disease, metastatic solid tumor, AIDS/HIV, and use of immunosuppressants).

Adjustment 3 was for the demographic characteristics of patients and chronic health condition, as previously mentioned, and location prior to ED admission (home, nursing home, and medical facility).

Abbreviation: qSOFA, quick sequential organ failure assessment; HR, hazard ratio; CI, confidence interval; ED, emergency department.

The association between prehospital qSOFA positivity and in-hospital mortality remained significant among the pre-specified subgroups of patients except for the subgroup of patients with respiratory infection. In 478 patients with respiratory infection, the association was not significant (adjusted HR: 1.67, 95% CI: 0.85–3.44; p = 0.14). The details of the subgroup analyses are shown in Table 3.

**Table 3:** Hazard ratios for in-hospital mortality in patients who are positive for prehospital qSOFA stratified as pre-specified subgroups.

	Number of case	HR (95% CI)	p value
**Age**			
< 75 years	308	3.98 (1.49–12.57)	<0.01
≥ 75 years	617	1.85 (1.06–3.38)	<0.05
**Sex**			
Male	469	1.94 (1.03–3.80)	<0.05
Female	456	3.49 (1.59–8.51)	<0.01
**Chronic health condition**			
Yes	546	2.09 (1.22–3.73)	<0.01
No	379	2.36 (1.04–5.84)	<0.05
**Location prior to ED admission**			
Home	546	2.06 (1.11–3.94)	<0.05
Nursing home or medical facility	379	3.42 (1.55–8.74)	<0.01
**Site of infection**			
Respiratory	478	1.67 (0.85–3.44)	0.14
Others	447	3.30 (1.66–7.09)	<0.01

Subgroup analyses were performed with the Cox proportional hazard regression model and in-hospital mortality censored at the discharge day. The demographic characteristics of patients, chronic health condition, and location prior to ED admission were incorporated into the multivariable models except for each stratification variable.

Abbreviation: qSOFA, quick sequential organ failure assessment; HR, hazard ratio; CI, confidence interval; ED, emergency department.

## Discussion

In this single center retrospective cohort study of 925 ED patients with suspected infection, a strong association was observed between prehospital qSOFA score and in-hospital mortality (adjusted HR: 2.41, 95% CI: 1.51–3.98; p <0.01). The association remained consistent between prehospital qSOFA and other outcomes (28- and 90-day mortality) and among various subgroups.

Currently, several studies have investigated the performance of the prehospital qSOFA score [15–17, 23–26]. Vaittinada Ayar P et al. have reported that in-hospital mortality rate was significantly higher in patients with prehospital qSOFA positivity among 332 patients suspected with infection (prehospital qSOFA-positive: 41% [55/133] vs prehospital qSOFA-negative: 18% [36/199], p<0.001) [24]. Shu E et al. have evaluated the prehospital qSOFA score of patients who were brought by EMS personnel and analyzed the prognostic value of the prehospital qSOFA score among 428 patients diagnosed with infection. They showed that an increase in prehospital qSOFA score was associated with in-hospital mortality (positive likelihood ratio 3.99, 95% CI: 2.21–7.21) [25]. In both of these studies, no covariate adjustments were made.

Our study results were in accordance with those of prior studies and have validated the association between the prehospital qSOFA score and mortality rate using the Cox proportional hazard regression model with covariate adjustments. We assessed the proportional hazard assumption in the primary analysis. Our study had a larger sample size (925 patients) than prior studies. In addition, subgroup analyses confirmed that the association between the prehospital qSOFA score and in-hospital mortality was consistent across different subgroups. In patients with respiratory infection, qSOFA might overly assess the risk of mortality. Most patients with respiratory infection had elevated respiratory rate and thus had increased qSOFA scores due to respiratory infection regardless of severity (adjusted HR: 1.67, 95% CI: 0.85–3.44; p = 0.14). Importantly, 6.0% (27/452) of prehospital qSOFA-negative patients died in our study. Although the prehospital qSOFA score had a significant association with mortality among patients with suspected infection, it had low sensitivity for mortality [5, 27–30].

The need to recognize infected patients in the prehospital setting was more and more emphasized. In April 2018, the Surviving Sepsis Campaign Task Force published the new revision of the sepsis bundle (hour-1 bundle), which recommends the complete initiation of resuscitation and treatments of patients with sepsis within 1 hour from the time of triage in the ED [31, 32]. Once EMS providers identified patients with infection before their arrival at the hospital, prehospital qSOFA score was an effective tool in estimating the mortality rate.

Our study had several limitations. First, it was a single-center, retrospective study. Therefore, the results cannot be generalized. Second, due to the difference between JCS and GCS, a GCS score < 15 may be misclassified as JCS 0, such as that in a patient who had good verbal and motor response without any stimulation but who was unable to open his/her eyes. However, this misclassification was a bias that acts on reducing the mortality difference between the prehospital qSOFA-positive and qSOFA-negative groups. However, the association between the prehospital qSOFA score and in-hospital mortality was still observed. Finally, we did not compare the prehospital qSOFA score with other severity or prehospital screening tools.

## Conclusions

Among the various subgroups of patients with suspected infection who were admitted in the ED, a strong association was found between the prehospital qSOFA score and in-hospital mortality. In order to use this score in clinical practice, future study is necessary to evaluate how infection is suspected in the prehospital arena.

## Supporting information

S1 Table Demographic data of the patients and characteristics that include laboratories, site of infection, and type of organisms. The prehospital qSOFA score was assessed using the first vital sign obtained at the scene and taken by EMS providers.
Abbreviation: qSOFA, quick sequential organ failure assessment; IQR, interquartile range; PT-INR, prothrombin time-international normalized ratio; Na, serum sodium; K, serum potassium; CRP, C-reactive protein; GNR, gram-negative rods; GNC, gram-negative cocci; GPC, gram-positive cocci; GPR, gram-positive rods; EMS, emergency medical service.
(PDF)
