# Supplementary material for: Use of prehospital qSOFA in predicting in-hospital mortality in patients with suspected infection: A retrospective cohort study
Source: PLoS One. 2019 May 7;14(5):e0216560. doi: 10.1371/journal.pone.0216560 (PMC6504075; doi:10.1371/journal.pone.0216560)
Supplement: S1 Table — The prehospital qSOFA score was assessed using the first vital sign obtained at the scene and taken by EMS providers. Abbreviation: qSOFA, quick sequential organ failure assessment; IQR, interquartile range; PT-INR, prothrombin time-international normalized ratio; Na, serum sodium; K, serum potassium; CRP, C-reactive protein; GNR, gram-negative rods; GNC, gram-negative cocci; GPC, gram-positive cocci; GPR, gram-positive rods; EMS, emergency medical service. (PDF) [file pone.0216560.s001.pdf]

S1 Table. Demographic data of the patients and characteristics that include laboratories, site of infection, and type of organisms

	prehospital qSOFA negative (<2) (N=452)	prehospital qSOFA positive (≥2) (N=473)	p value
**Laboratory (median [IQR])**			
Leucocytes, cells/ml	10,100 [7,300, 1,300]	10,700 [7,200, 14,700]	0.14
Hematocrit, %	36.3 [32.5, 40.4]	35.9 [31.4, 40]	0.12
Platelet, 10^3/μl	18.9 [14.7, 24.8]	19.2 [14.3, 26.0]	0.56
PT-INR	1.08 [1.02, 1.18]	1.13 [1.04, 1.25]	<0.01
Na, mEq/l	136 [132, 138]	135 [131, 139]	<0.05
K, mEq/l	3.9 [3.5, 4.3]	4.1 [3.7, 4.5]	<0.01
Total bililbin, mg/dl	0.7 [0.5, 1.1]	0.65 [0.4, 1]	0.10
Creatinin, mg/dl	0.76 [0.59, 1.1]	0.77 [0.54, 1.18]	0.87
CRP, mg/dl	4.52 [1.25, 11.09]	5.20 [1.78, 11.99]	0.10
Glucose, mg/dl	121 [105, 150]	125 [105, 158]	0.14
Lactate (mmol/l)	1.5 [1.1, 2.2]	1.8 [1.1, 3.3]	<0.01
**Site of infection (%)**			0.12
Bloodstream	13 (2.9)	16 (3.4)	
Respiratory	214 (47.4)	264 (55.8)	
Gastrointestinal	69 (15.3)	50 (10.6)	
Neurological	5 (1.1)	8 (1.7)	
Genitourinary	110 (24.3)	100 (21.1)	
Musculoskeletal	30 (6.6)	23 (4.9)	
Other	11 (2.4)	12 (2.5)	
**Type of organism (%)**			0.62
Not specified	217 (48.0)	229 (48.4)	
Escherichia coli	77 (17.0)	65 (13.7)	
Klebsiella pneumoniae	20 (4.4)	34 (7.2)	
Pseudomonas aeruginosa	10 (2.2)	10 (2.1)	
Other GNR	35 (7.7)	38 (8.0)	
GNC	8 (1.8)	9 (1.9)	
Staphylococcus aureus	5 (1.1)	7 (1.5)	
Streptococcus pneumoniae	11 (2.4)	10 (2.1)	
Streptococcus species	17 (3.8)	14 (3.0)	
Other GPC	10 (2.2)	5 (1.1)	
GPR	3 (0.7)	7 (1.5)	
Polymicrobial infection	39 (8.6)	45 (9.5)	
